# DNA in extracellular vesicles: from evolution to its current application in health and disease

**DOI:** 10.1186/s13578-022-00771-0

**Published:** 2022-03-28

**Authors:** Jamal Ghanam, Venkatesh Kumar Chetty, Lennart Barthel, Dirk Reinhardt, Peter-Friedrich Hoyer, Basant Kumar Thakur

**Affiliations:** 1 Department of Pediatrics III, University Hospital Essen, University of Duisburg-Essen, 45147 Essen, Germany; 2 Department of Neurosurgery and Spine Surgery, Center for Translational Neuro- and Behavioral Sciences, University Hospital Essen, 45147 Essen, Germany; 3 Institute of Medical Psychology and Behavioral Immunobiology, Center for Translational Neuro- and Behavioral Sciences, University Hospital Essen, 45147 Essen, Germany; 4 Department of Pediatrics II, University Hospital Essen, University of Duisburg-Essen, 45147 Essen, Germany

## Abstract

Extracellular vesicle (EV) secretion is a highly conserved evolutionary trait in all organisms in the three domains of life. The packaging and release of EVs appears to be a bulk-flow process which takes place mainly under extreme conditions. EVs participate in horizontal gene transfer, which supports the survival of prokaryotic and eukaryotic microbes. In higher eukaryotes, almost all cells secrete a heterogeneous population of EVs loaded with various biomolecules. EV secretion is typically higher in cancer microenvironments, promoting tumor progression and metastasis. EVs are now recognized as additional mediators of autocrine and paracrine communication in health and disease. In this context, proteins and RNAs have been studied the most, but extracellular vesicle DNA (EV-DNA) has started to gain in importance in the last few years. In this review, we summarize new findings related to the loading mechanism(s), localization, and post-shedding function of EV-DNA. We also discuss the feasibility of using EV-DNA as a biomarker when performing a liquid biopsy, at the same time emphasizing the lack of data from clinical trials in this regard. Finally, we outline the potential of EV-DNA uptake and its interaction with the host genome as a promising tool for understanding the mechanisms of cancer evolution.

## Highlights

- Protecting DNA in membrane vesicles seems to be a conserved phenomenon for the horizontal genetic flux between prokaryotes and lower eukaryotes.
- Capturing and analyzing this vesicular DNA enables quick and non-invasive monitoring of natural ecosystems.
- Cancer-derived extracellular vesicles containing DNA open up novel directions in cell-to-cell communication and therefore disease monitoring.
- Complex and fluctuating conditions of the tumor microenvironment, mimicking natural ecosystems, could favor EV-DNA release, mediating tumor multi-clonal evolution and providing survival benefits.

## Introduction

Cells receive and coordinate multiple information and signals among themselves, which finally determines the fate of an organism. Through this process, cells from one compartment will either proliferate, migrate to other compartments for some physiological function, or undergo apoptosis. Malignancy occurs when there is an imbalance in tissue homeostasis in which the natural symbiosis between cellular and microenvironmental components is disturbed. In particular, tumorigenesis occurs when normal cells accumulate DNA damage and irreversible changes known as mutations owing to many intrinsic and extrinsic factors. Cells can undergo up to one million DNA changes per day, including the integration of external or foreign DNA [1]. This integration mostly occurs when DNA derived from another organism of the same or a different species gains entry into the host cells [1].

It has long been known that extracellular DNA (exDNA, see Box 1 for more about the terminology used in this review), including ancient DNA, is responsible for lateral/horizontal gene transfer (HGT) in microbial ecosystems [2–4]. In 1911, Francis Peyton Rous demonstrated that cancer could be transmitted through cell-free tumor extracts, and to a lesser extent via a small transmissible agent such as a virus [5]. This was the first evidence of HGT between submicroscopic infectious agents and higher eukaryotes [5]. Human oncogenic viruses contribute to 15–20% of all human cancers [6] by causing direct insertional mutagenesis or by manipulating the signaling pathways that monitor and repair DNA damage during and upon replication [6, 7]. The mechanisms of exDNA uptake and how exDNA overcomes cell defense and integrates into the genome are yet to be clarified.

**Fig. 1:**
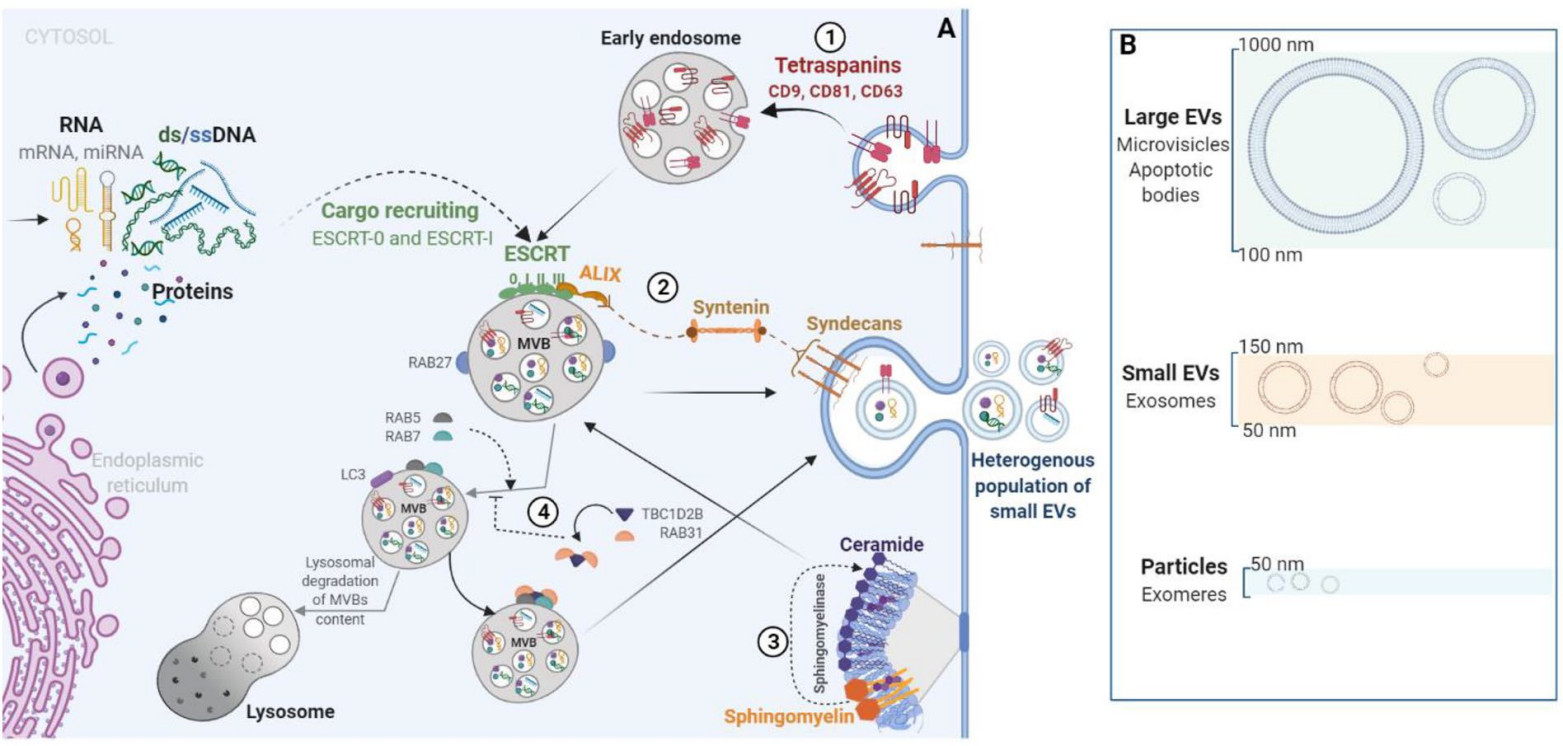
Extracellular vesicles release is a highly organized and conserved phenomenon. **b** Cells release a wide range of extracellular vesicles, including large EVs like microvesicles and apoptotic bodies, small EVs known as exosomes, and small particles (< 50 nm) recently named exomeres. **a** Up to date biogenesis pathways of small EVs: **1** tetraspanin-enriched microdomains; **2** Sendycan-syntenin-ALIX: The cytosolic adaptor syntenin reacts with sendycan through its PDZ domains and with ALIX via three LYPXnL motifs. In turn, ALIX binds to ESCRT-III, which promotes intraluminal vesicle budding. ESCRT-0 and ESCRT-I recruit the small EVs cargo, while Rab27 helps the multi-vesicular body (MVB) fusion with the cell membrane; **3** MVB could be directed to lysosomes for degradation with the help of RAB7, RAB5, and the autophagy-related protein LC3. As a new ESCRT-independent pathway for small EVs biogenesis, RAB31 has been found to recruit GTPase-activating protein (TBC1D2B) that inactivates RAB7 and thereby favors EVs content secretion rather than the degradation in lysosomes. **4** Sphingomyelinase catalyzes the conversion of sphingomyelin to ceramide and phosphorylcholine. Ceramide enriched endosomes tend to form inward buds ending in ILV formation

In both normal and pathological conditions, cells release tiny vesicles known as extracellular vesicles (EVs) across the extracellular space. It is well known that EVs pass the information from one cell to another, cell-to-cell communication that is important in both health and disease. Cells release different types of EVs which differ in size, shedding mechanism, and function (Fig. 1). Many researchers have shown that cancer-derived EVs contain DNA (EV-DNA), which reflects the mutational status of parental cancer cells [8, 9]. It is still not understood why and how cells communicate via EV-DNA and what the functional role of this communication is, especially in cancer biology. In this review, we explore the role of EV-DNA in cancer evolution considering the lessons learned from prokaryotes and eukaryotes, in which DNA has played a crucial role in triggering the evolutionary process for millions of years. We believe that understanding the biology of EVs and EV-DNA from an evolutionary point of view would enable us to better understand their normal biological functions and resilience.

Box 1: Terminology In literature, the terms cfDNA and exDNA are often used to define DNA outside the confinement of cells as opposed to intracellular DNA (inDNA). In contrast, the term eDNA is chiefly used to determine exDNA found in environmental samples such as sand, soil, or water. cfDNA and exDNA are often used interchangeably to describe any form of DNA found in the interstitial space, circulation, or body fluids. This includes mitochondrial DNA (mtDNA), DNA associated with extracellular vesicles, foreign DNA, naked and free ambient DNA molecules, and DNA associated with protein complexes released actively or passively from host and microbial cells. DNA associated with EVs has long been considered part of cfDNA. For the reader’s convenience we retain the abbreviations gDNA for genomic DNA, eDNA for environmental DNA, cfDNA for cell-free DNA, exDNA for extracellular DNA, and EV-DNA for extracellular vesicle DNA.

## HGT: from Darwin’s “gemmules” to bacterial membrane vesicles

HGT is the transfer of genetic information between organisms other than parent-to-offspring inheritance, which may occur via plasmids, transposons, viruses, or other unknown vectors*.* In 1868, Charles Darwin proposed the Pangenesis theory, in which he evoked HGT within organisms. Interestingly, he suggested that all cells in an organism are capable of shedding “minute particles” which he named “gemmules” [10]. These circulating self-replicating entities were assumed to cogenerate the gonads, allowing offspring to inherit information. Darwin assumed that these particles travel through the body and vary in response to the individual’s environment [10]. He supposed that this process might be another form of unexplored cellular function in which the protected "genetic information" in “gemmules” is horizontally transferred to germ cells. Although the Pangenesis theory sounds philosophical and has been widely rejected by the scientific community, the idea of transporting DNA in a membrane case is still attractive—what if Darwin's “gemmules” were EVs associated with cell-free DNA?

## Membrane vesicles are bacterial “gemmules”

In 1928, Frederick Griffith discovered that virulent traits could be transferred from heat-killed bacteria to non-pathogenic strains in a culture medium [11]. Following this observation, in 1944 Oswald Avery demonstrated in an experiment that acquisition of pathogenic properties is due to the transfer of cell-free genetic material "of the desoxyribose type" (DNA) [12]. Over the past few decades, and with the advent of new technologies in genome sequencing, it has been unambiguously demonstrated that HGT is a major driving force that has constantly reshaped genomes throughout evolution [13]. Microbes receive and integrate DNA from a variety of sources, including extracellular vesicles [14–17]. Bacteria release different types of extracellular vesicles known as membrane vesicles (MVs), schematized in Fig. 1 and extensively reviewed elsewhere [18, 19]. Further the shedding rate of MVs increases under antibiotic stress and complex biotopes such as biofilms, rumen, and marine ecosystems [16, 17, 20].

Box 2: EV-DNA mediates clinical evolution of the human malaria parasite MVs participate in the genetic flexibility of prokaryotic microbes, but little is known about gene transfer via MVs in eukaryotic microbes. Recently, studies have focused on genetic communication via vesicles in the malaria parasite *Plasmodium falciparum*. Sundararaman et al. indicated a possible emergence of the human parasite *P. falciparum* through DNA exchange from an ancestor of a gorilla parasite, *P. adleri*, to the ancestor of another gorilla parasite, *P. praefalciparum* [21]. The authors suggested that these *P. adleri* > *P. praefalciparum* > *P. falciparum* “evolutionary transfer events” were likely achieved through the asexual transfer of small amounts of DNA during the bloodborne infection stage, mainly via EVs [21]. Indeed, this suggestion was based on previous findings reporting that *P. falciparum* can be transformed by the spontaneous take-up of erythrocytes’ cytoplasmic DNA [22]. More interestingly, infected erythrocytes communicate via microvesicles and exosome-like vesicles, which promote differentiation to the sexual form of the parasite [23, 24]. Additionally, exosome-like vesicles derived from erythrocytes upon infection with transgenic *P. falciparum* are able to deliver DNA encoding for a drug resistance marker and can support the growth of normal *P. falciparum* under conditions of drug selection [24].

MVs have been involved in intra- and inter-species transfer of DNA encoding for many vital characters, including antibiotic resistance and metabolic genes [25–30]. DNA exchange via MVs occurs naturally in complex environments, as Biller et al. demonstrated by studying MVs released by the cyanobacterium *Prochlorococcus* [20]. Intriguingly, *Prochlorococcus* MVs are able to support the growth of heterotrophic bacteria, which implies that they are involved in the carbon cycle in the marine ecosystem, probably owing to the transfer of metabolic genes [20]. Hence, MVs may be released when communication via soluble messengers is limited, suggesting the possible use of MVs as a "primitive" form to share information within and across species. This genetic communication allows microorganisms to acquire new hereditary characters (virulent, resistant, and metabolic traits) influencing their behavior in an evolutionary manner (not a transient effect). In the following sections, we elucidate the possibility of genetic communication through EV-DNA in higher eukaryotes. However, it has not yet been explicitly proven whether EVs are carrying entire and functioning genes.

## Genetic communication via EV-DNA

### The varied nature of EV-DNA

Until 2014, only single-stranded DNA (ssDNA), mitochondrial DNA (mtDNA), and repetitive transposons were described as being in EVs, including exosomes [31, 32]. These studies concluded the presence of DNA in EVs based on enzymatic methods, digesting DNA associated with EVs with the help of S1 nuclease to demonstrate the presence of ssDNA, or with DNase I to claim the presence of double-stranded DNA (dsDNA). In 2014, Kahlert et al. and Thakur et al. provided the first evidence of dsDNA in cancer exosomes [8, 9]. The work of Kahlert et al. (2014) used the DNase I-based digestion approach to show the existence of genomic DNA in exosomes derived from pancreatic cancer cells. Biochemically, DNase I is an endonuclease that non-specifically digests both single- and double-stranded DNA. Therefore, employing an alternative method, Thakur et al. (2014) utilized a double enzymatic approach by including S1 nuclease that digests ssDNA and a shrimp dsDNase that specifically hydrolyzes dsDNA. This work showed that the majority of gDNA associated with exosomes is dsDNA. Further, dsDNA can be a diagnostic marker of the molecular properties of a primary tumor in vitro and in vivo [9]. This approach has been applied in multiple studies to ensure that only internally packaged DNA is characterized and to confirm the nature of DNA associated with the EVs as either ssDNA or dsDNA [33, 34].

### Origin and packaging of EV-DNA

**Fig. 2:**
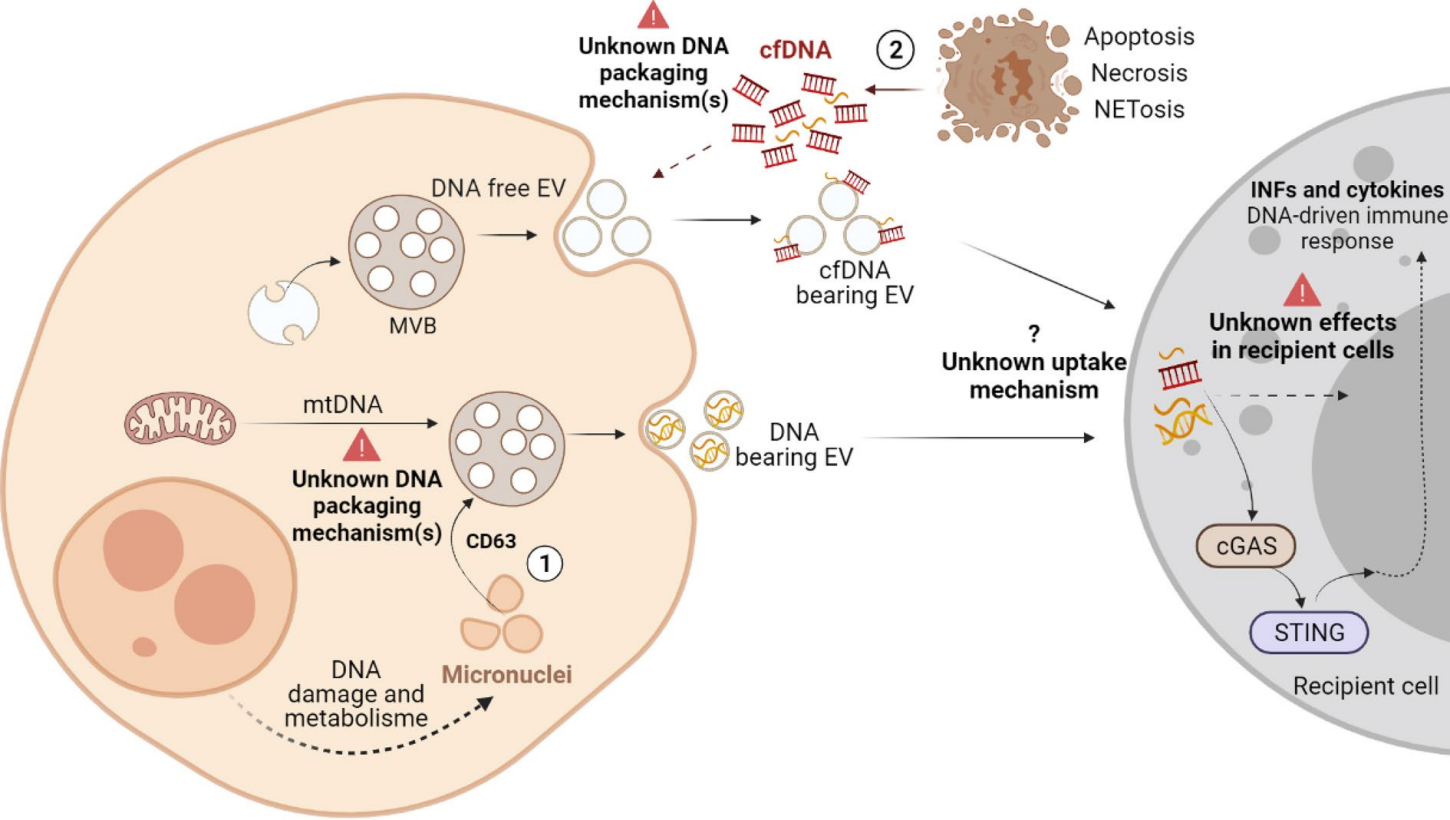
The putative origin of EV-DNA. The exact mechanisms of EV-DNA packaging remain obscure. **1** In micronucleated cells, EV-DNA loading may occur in the cytoplasm via the endosomal tetraspanins like CD63. **2** cfDNA released by dead cells could also be a source of EV-DNA by sticking on the surface of released EVs. Upon uptake, EV-DNA induces the expression of pro-inflammatory cytokines through the so-called cGAS/STING pathway. Figure generated in Biorender

Both specific packaging and distinct sequences of EV-DNA indicate that this DNA is destined to be used outside the origin cell, which opens up many questions about the mechanism(s) by which this DNA comes into EVs and its function(s) outside the cell. We can only speculate at this point, but some explanations concerning DNA loading into EVs are emerging. Thus, we will discuss what is known so far in this nascent field, and we want to initiate a discussion that could lead us to reveal the secret behind EV-DNA packaging and its function (Fig. 2).

While the existence of DNA in EVs is now considered a consensus in the field of EVs, the variability of this DNA and its packaging mechanisms remain unclear. EV-DNA could either be of nuclear (ncDNA) [8, 9] or mtDNA origin [32, 35], and ranges from small fragments of around 100 bp to fragments exceeding 10 Kbp. Interestingly, Sansone et al. demonstrated that the presence of ND1 mtDNA in circulating EVs appears specifically in patients with hormonal therapy-resistant breast cancer and is not simply a reflection of metastatic disease burden [35]. mtDNA has been found in EVs enriched from the plasma of atrial fibrillation [36] and chronic heart failure patients [37]. Accumulating observations also imply that EV-DNA includes DNA from retrotransposable elements and satellite repeat DNA [31, 38]. In particular, the recent finding of Cambier et al. (2021) confirmed that repetitive element DNAs, such as HSATI, HSATII, LINE1-P1, and Charlie 3, were associated with EVs derived from the serum of osteosarcoma patients but not with CD9+ or CD81+ exosomes [38].

It has long been thought that EV-DNA mainly comes from cytoplasmic DNA fragments that resulted from either regular DNA metabolism or DNA damage [39, 40]. However, this hypothesis does not sufficiently explain the above-mentioned heterogeneity of EV-DNA. A few recent studies have provided insights into this area, but always in the context of cytoplasmic DNA excretion. In 2017, Takahashi et al. reported that loading of harmful cytoplasmic DNA into exosomes (small EVs) maintains cellular homeostasis and avoids senescence-like cell-cycle arrest or apoptosis [41]. In particular, the inhibition of exosomes secretion has been found to activate the cytoplasmic inflammatory sensing machinery, cGAS/STING, owing to accumulation of nuclear DNA fragments in the cytoplasm [41]. In addition, Harding et al. showed that micronuclei (MN), small cytoplasmic budding of the nucleus resulting from missegregation of nuclear material during mitosis or DNA damage (e.g., in response to genotoxic treatment), promote the activation of the cGAS/STING pathway [42]. This results in an inflammatory response against both senescent and cancer cells [42]. More recently, Yokoi et al. identified a relationship between MN formation and nuclear exosome secretion upon induction of genomic instability by genotoxic drugs [43]. Their findings suggest that the extremely unstable membrane of MN collapses upon treatment with genotoxic drugs. Additionally, the nuclear contents of MN released into the cytoplasm are packaged into the microvesicular bodies in the tetraspanin (CD63)-based endosomal sorting pathway and released into extracellular space in the form of exosomes [43]. On the one hand, this could to some extent explain the loading of cytoplasmic DNA into EVs via the endosomal sorting complex required for transport (ESCRT) pathway, while the packaging of mtDNA and other DNA types is still a conundrum. On the other hand, in both studies, small EVs were enriched using crude, traditional ultracentrifugation known to co-isolate many contaminants as well as heterogeneous EV populations. This implies the need for further research to confirm the same effects using homogenous vesicle populations.

### Extracellular vesicle uptake by eukaryotic cells

EVs can transfer information and exert their effects by directly binding to surface receptors or by fusion to recipient cell membranes, which triggers intracellular signaling pathways and allows the complete uptake of EV content. EVs can also be taken up by recipient cells via different mechanisms, including endocytosis, clathrin-coated pits, lipid rafts, phagocytosis, caveolae, and micropinocytosis [44]. However, it is not yet clear which of these mechanisms is responsible for the uptake of EVs containing DNA. Using immunofluorescence, Cai et al. showed that acridine orange-stained EV-DNA could be transferred into recipient cells and localize to the inside of the nuclear membrane [45]. Interestingly, Fischer et al. demonstrated that bone marrow mesenchymal stromal cells transfected with EVs containing exogenous DNA (from *Arabidopsis thaliana*) are shown to pass their stably integrated EV-DNA to three generations of daughter cells [33]. Furthermore, possible trafficking of MV-derived DNA through the cytosol and to the nucleus or perinuclear space has been suggested in non-phagocytic A549 eukaryotic host cells (adenocarcinoma human alveolar basal epithelial cells) [16]. The CRISPR-Cas9 system, another lesson learned from lower organisms, is nowadays considered the method of choice for genome editing. CRISPR-Cas9-based genome editing starts by generating a double-strand break (DSB) and subsequent cellular DNA repair process. The resulting DNA DSB is repaired either by the error-prone nonhomologous end joining or homology-directed repair [46]. Ono et al. have reported that HGT can occur during DSB repair via DNA from small EVs [47]. This indicates that the unintentional capture of EV-DNA sequences at DSB sites might be an evolutionary driving force of mammalian genomes [47].

## How EV-DNA is introduced into the nucleus?

The above-mentioned studies demonstrating the possibility of EV-DNA uptake and integration into the host genome, although intriguing, require validation in the context of their relevance in cell communication in health and disease. As we have previously discussed, only a distinct subpopulation of EVs contains DNA. It appears counterintuitive that this set of EVs fuses directly with the cell membrane and releases the DNA cargo into the cytoplasm, since naked DNA will be prone to degradation and excretion via the DNA cytoplasmic sensing machinery [48]. Hence, this mechanism requires further investigation [49]. Instead, it is possible that DNA-associated EVs may fuse directly with the nuclear membrane after internalization, which may protect EV-DNA against a cytoplasmic inflammatory response or by escaping the endosomal-lysosomal degradation route. It would seem that some viruses are using this mechanism to avoid immune-mediated clearance. In fact, EVs from HCV-infected human hepatoma Huh7.5.1 cells contain full-length viral RNA, viral protein, and particles [50]. Feng et al. found that the hepatitis A virus released from cells is cloaked in host cell-derived exosomes, thereby protecting the virion from antibody-mediated clearance [51]. Saari et al. have recently suggested that delivering oncolytic viruses in EVs helps avoid immune system, which constitutes an alternative entry pathway into cancer cells [52]. The use of the host’s vesicle machinery to escape the immune system is another lesson we should learn about the importance of DNA packaging into EVs. In addition, similarities in size and density between small EVs and viruses may open up new paths to understanding some of the secrets behind EV-DNA uptake and integration.

## EV-DNA in cancer diagnostics

### Tumor heterogeneity and diagnostics

Comparable to natural ecosystems (Box 3), interactions within tissues and organs generate a complex environment in multicellular organisms. Tumorigenesis has been recognized as an evolutionary process that is driven by the same Darwinian forces that drive the evolution of species in natural ecosystems [53–58]. Nonetheless, not all evolutionary principles are applicable, and the divergence in DNA sequences is minimal in cancer (evolutionary distance of ~ 10^−6^ or ~ 1 bp per Mb of single nucleotide change) [57]. A tumor can evolve from one cell that accumulates genetic changes (either driver or passenger mutations) in response to environmental forces. These changes enable tumor cells to acquire new traits over their normal counterparts, allowing them to resist therapy and disseminate to distant locations from the primary tumor site. For example, colorectal tumors evolve through a succession of mutations starting in the APC gene in the slowly growing adenoma, followed by clonal expansion when cells accumulate new mutations in genes such as KRAS, PIK3CA, SMAD4, and TP53 [59]. Simultaneously, epigenetic forces such as differential access to nutrients and oxygen, immune system responses, and drug pressure promote tumor evolution, which results in highly aggressive and heterogeneous multi-clonal phenotypes [60].

**Fig. 3:**
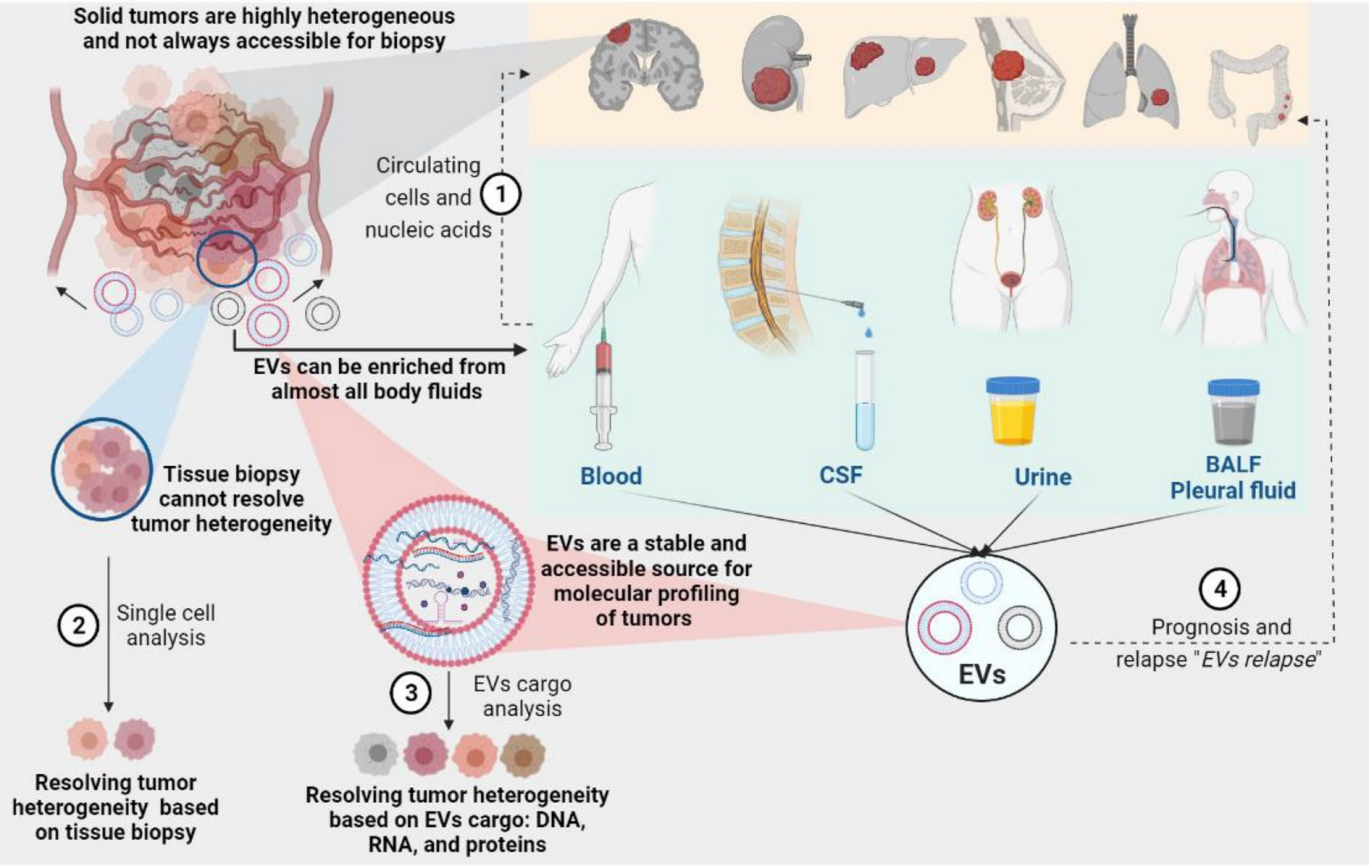
Extracellular vesicles and their DNA are promising tools for liquid biopsy. **1** Analysis of circulating tumor cells and nucleic acids (ctDNA and ctRNA) is being installed in clinical settings for cancer diagnosis as it has recently been discussed by Ignatiadis et al. (2021), who discussed the notion of “ctDNA relapse” [106]. Circulating tumor cells split away from the primary tumor and constitute the seed of metastases, limiting their use for cancer prognosis and relapse [63]. **2** Single-cell sequencing (along with omics analyses) is the method to study tumor heterogeneity. This depends mainly on the number of analyzed cells determined by the quality of tissue biopsy. **3**, **4** stable and well-protected DNA, RNA, and proteins can be repeatedly enriched from EVs found in almost all body fluids. Analysis of EVs cargo can provide a dynamic genome and metabolome landscape for a real-time assessment of disease evolution and relapse: “EVs relapse.”

Rudolf Virchow reported tumor heterogeneity as early as the nineteenth century [61]. Intra-tumor heterogeneity and sub-clonal evolution of tumors lead to a spatiotemporal variability of physiological determinants, which can be used as important clinical biomarkers and can help with diagnosis, treatment, and monitoring of therapies. This implies the necessity of repeated surveillance of the phenotypic variability of tumor sub-clones, an approach that has not yet been standardized in clinical routine. However, cancer monitoring and diagnostics still primarily rely on invasive biopsy or surgical specimens that consider cancer as a monoclonal disease (Fig. 3).

Such a diagnostic tool is particularly important for tumors that are not suitable for a biopsy [62]. To date, standard histological methods rely on invasive biopsies that are often carried out once and not in a frequent sequence. Approaches based on cell-free DNA, single-cell sequencing (SCS), and single-cell multi-omics have been so far employed for deciphering clonal evolution in cancer. A typical SCS experiment goes, however, through sampling (biopsy), cell sorting (physical separation of each individual cell), whole genome or transcriptome amplification, library construction, sequencing, and data analysis [63]. Here again, one of the most prominent challenges in SCS is the amount of cells that need to be sequenced for significant representation of the tumor landscape. This would depend on the quality of the specimen and whether it is covering all tumor sub-clones.

Box 3: Extracellular DNA and EV-DNA: an established tool to monitor natural ecosystems Biomonitoring in natural ecosystems has long relied on direct visual surveys of macro- and micro-organisms in the field [64]. These traditional methods and practices are still far from being conclusive in explaining the spatiotemporal evolution of ecosystems, mainly resulting from increasing modern human activities such as pollution, which interferes directly with natural ecosystems. However, since the discovery of environmental DNA in 1987, advances in metagenomic and DNA metabarcoding approaches have fundamentally revolutionized biodiversity surveys and ecosystems monitoring [65–69]. Conceivably, eDNA may include gDNA, exDNA, mtDNA, and EV-DNA (Box 1) since it is enriched from the environment without a pre-isolation of target organisms. Continuous release of DNA in complex natural and dynamic ecosystems results from intra- and inter-species interactions and organisms’ responses to biotic and abiotic factors and stressors. High-throughput generation of data directly from eDNA extracted from water, soil, sediments, or air samples enables a continuous spatiotemporal assessment of biodiversity in a *non-invasive manner* [65]. Additionally, advanced computational tools allow the study of historical changes and long-term predictions of natural ecosystem fluctuations, such as the postglacial viability and colonization in North America by humans [70]. The fact that eDNA is a tool for recording and identifying ecological and evolutionary changes in macro-ecosystems is now beyond doubt.

## EV-DNA or cfDNA for performing a liquid biopsy

Cell-free DNA (cfDNA) has emerged as a promising biomarker for cancer diagnosis and prognosis [71]. Depending on its size and configuration (single or double stranded), cfDNA half-life in the circulation ranges from 15 min to 1–2 h before being degraded and cleared. This occurs in tissues, blood or other body fluids, and organs (liver, spleen, kidney, or lymph nodes) [72]. cfDNA is highly fragmented, complicating its analysis using methods associated with next-generation sequencing (NGS) [73, 74]. Furthermore, cfDNA originates from cells undergoing apoptosis or necrosis, which may not reflect the viable cell population of the tumor [75]. By contrast, EV-DNA is released by metabolically active cells, which may represent different tumor sub-clones. In addition, EV-DNA is protected inside a lipid bilayer, making it a more stable and representative source to characterize a tumor's microenvironment heterogeneity. The detection of mutations associated with cancer cells and tumors in EV-DNA has further underscored the utility of EV-DNA in cancer diagnosis [8, 9]. Indeed, despite early skepticism about the specificity of DNA detection in vesicles, many studies (Table 1) have shown the advantage of EV-DNA (ss/dsDNA) for the detection of mutations in cancer. This opens a promising avenue toward performing an easy and reliable "liquid biopsy" in patients. Table 1 highlights the future perspective of using EV-DNA in a liquid biopsy to obtain information about the cancer status.

**Table 1:** Potential use of EV-DNA as a biomarker for cancer

Disease	EVs source	Isolation method	EVs/DNA types	Mutation	References
Cancer					
Melanoma	Cell lines preclinical mice model	UC	exo/dsDNA	EGFR and BRAF	[9]
Pancreatic cancer	Plasma	UC	exo/dsDNA	KRAS, p53	[8]
Pancreatic cancer	Plasma	UC	exo/dsDNA	KRAS, p53	[90, 91]
Pancreatic cancer	Plasma	Microfluidic platform	ev*/DNA*	KRAS	[92]
Pancreatic cancer	Serum	UC	exo/DNA*	KRAS	[93]
Pancreatic cancer	Seum and plasma	ExoEasy Maxi Kit UC	exo/DNA*	KRAS	[94]
Glioma	Small cohort	UC	ev*/DNA*	Multiple mutations	[95]
Glioblastoma multiforme	Cell lines	PEG-NaCl precipitation	exo/DNA*	NANOGP8 ^a^	[96]
Osteosarcoma	Serum	PEG precipitation SEC	ev*/DNA*	Repetitive element DNAs	[38]
Acute myeloid leukemia	Plasma	UC	ev*/dsDNA		[97]
Urothelial bladder carcinoma	Clinical study/urine	ExoQuick-TC ^c^	exo/DNA*	Multiple mutations and CNVs ^b^	[98]
Non-small cell lung cancer	Plasma and bronchoalveolar lavage fluid	UC	ev*/DNA*	EGFR	[99]
Lung adenocarcinoma	Malignant pleural effusions	ExoQuick-TC ExoLution plus isolation kit	exo/dsDNA	EGFR	[100, 101]
Pheochromocytomas and paragangliomas	Serum preclinic	UC	exo/dsDNA	Multiple	[102]
Ovarian cancer	Plasma	miRCURY ™ kit ^c^	Exo/mtDNA	Copy number of mtDNA	[103]
Other conditions					
Tuberculosis	Clinical specimens	ExoQuick™	exo/DNA*	MTB-specific IS6110	[104]
Fetal trisomy	Plasma	ExoQuick™	ev*/DNA*	Trisomies, and de novo FGFR3 mutations	[105]

*Not identified

^a^An insertion of 22 bp of NANOGP8 gene into the 3′ UTR

^b^Copy number variation (CNV)

^c^Commercial exosome precipitation kit

Nonetheless, the clinical application of EV-DNA is still not yet validated. Over a hundred clinical trials are registered in ClinicalTrials.gov in order to investigate the use of EVs in clinical settings. Some of these studies have included EV-DNA as a cancer biomarker, such as clinical trial NCT03217266, which examines the use of the MDM2 inhibitor AMG-232 (KRT-232) and radiation therapy to treat patients with soft tissue sarcoma [76]. Another clinical trial (NCT04523389) aims to test the feasibility of EV-DNA (and other EV contents) as biomarkers in colorectal cancer patients [77]. Similarly, NCT03236675 demonstrates the feasibility of detection of EML4-ALK fusion transcripts and T790M EGFR mutation from small EVs in the circulation of non-small cell lung cancer (NSCLS) patients [78]. On the other hand, NCT03228277 is a single-arm, open-label, Phase 2 study that aims to assess the anti-tumor efficacy of Olmutinib (Olita^®^) administered to patients with T790M-positive NSCLC confirmed using DNA extracted from EVs derived from bronchoalveolar lavage fluid [79].

EV purity is the major limitation that needs to be addressed in many studies listed in Table 1. The majority of studies failed to show that the observed DNA is associated exclusively with EVs and not with other constituents such as free-floating DNA or other contaminant proteins. In many of the studies mentioned, EV preparation relied on commercial isolation kits, precipitation agents (such as polyethylene glycol), and differential ultracentrifugation known to co-isolate many other contaminants along with EVs. This constraint can be overcome by using EV isolation methods that provide greater purity in the future. Targeted capturing of only the DNA-containing EV fraction among different EV populations is also a major challenge, mainly in clinical settings. This approach requires a complete understanding of the mechanisms and circumstances of EV shedding to ultimately define their role(s) in tumorigenesis and tumor evolution. Therefore, enriching only DNA-containing EVs will lead to an increase in the sensitivity of cancer mutational analyses, even at microscopic clone level or very early stages of cancer relapse or metastasis.

Conventionally, the molecular content of EV fractions is studied by western blot, omics technologies, or other bulk analyses that do not discriminate between single EVs. Thus, the information that can be obtained about EV fractions mainly depends on the purity of the samples under study. Dynamic light scattering, nanoparticle tracking analysis, and resistive pulse sensing are techniques frequently used for the quantification and size estimation of EVs [80–82]. Unfortunately, these technologies cannot detect and analyze the presence of biomolecules on or within individual EVs. Antibody-based immune gold labeling of EV components and imaging with transmission electron microscopy is currently the only established technology allowing single EV analyses. However, this method is time-consuming and can analyze only a limited number of antigens. It can hardly be used for quantitative EV analyses and qualitative EV capturing. A better understanding of the heterogeneity of EVs, when derived from a mixture of healthy and cancerous cells (in the blood plasma of cancer patients), could be helpful to obtain EVs with biomarker potential and functional relevance.

## Potential clinical significance of EV diagnostics in the future

The prevalence of cancer and other chronic diseases is expected to increase worldwide in the coming years. Disease prevention relies on primary and secondary approaches. The primary approach aims to minimize the risk factors by adopting a better diet and lifestyle habits. The secondary approach aims to detect disease at earlier and more curable stages. Current screening, diagnosis, therapy, and aftercare concepts are technically complex and require a high level of specialist personnel who work on-site near the patient. However, the strongest population growth will be seen in Africa and parts of Asia, thus in regions of the world where technical and human resources will probably not be available in sufficient quantities to meet the demand. Therefore, diagnostic and therapeutic strategies are required that are not very costly, require few human resources, and can also be carried out over greater distances. Diagnostic methods based on liquid biopsy represent a method that could meet these requirements. Blood, saliva, urine, or cerebrospinal fluid samples can be taken and examined far away with less effort. cfDNA- or EV-based diagnostic methods are very promising. cfDNA-based methods are already partially established in clinical areas such as perinatal diagnostics [83]. A recent proteomics profiling of 426 human samples from tissue explants, plasma, and other body fluids identified new EV biomarkers for pancreatic and lung cancer diagnosis and treatment [84]. As discussed above, authors have also mentioned the heterogeneity of EVs and protein contamination as a limitation and suggest combining genotyping tests based on circulating DNA and RNA for more accuracy [84, 85].

In the future, not only will genetic examinations play an essential role, presumably in connection with freely circulating DNA, but also examinations of proteins, inflammatory messenger substances, or lipids. All these factors can be examined in extracellular vesicles and might be valuable for more precise diagnostics. It may be possible to replace established and complex screening methods, therapy control, or follow-up care. Since EVs are not only a separate, passive product of maternal cells but are also intercellular mediators/communicators, it is conceivable to use EVs as a therapeutic vehicle. The potential applications are diverse and not limited to newly developing diseases.

## Conclusion and future directions

Although EV-DNA is now an essential part of the dynamics and survey of natural ecosystems, its role is largely overlooked in the realm of cancer biology and in monitoring the tumors microenvironment. The EV-DNA field is scarcely being explored, and lack of data, compared with research on EV protein and RNAs, is undoubtedly a critical constraint in fully understanding the mechanism(s) behind loading DNA into EVs. Yet, the EV field lacks a defined nomenclature, as new technological advances in EV isolation identify novel EV populations and can distinguish non-EV particles which were previously undetectable [39, 84, 86]. Packaging and shedding mechanisms beyond the ESCRT-dependent pathway remain to be explored in the future [87]. However, it has recently been found that RAB31 marks and controls an ESCRT-independent exosome biogenesis pathway, while the differential release of small EVs by polarized epithelial cells is controlled by ALIX and ceramide [88, 89]. This opens up a broad range of questions and concerns regarding the packaging of EV content which will undoubtedly include EV-DNA (see the Outstanding Questions in Box 4). In addition, we need to know more about the kinetics of EV-DNA packaging and of the EV release pattern during a single cell cycle, as well as different factors (intrinsic and extrinsic) affecting this process. Finally, EVs can be found in almost all body fluids, and EV-DNA seems to be more stable compared with cfDNA owing to protection by the lipid bilayer. The development of a system for rapid characterization of active EV-DNA (with specific mutations) could open up the possibility of performing repeated liquid biopsies in patients.

Box 4: Outstanding questions How do we distinguish DNA which is passively released by dying cells in the form of apoptotic bodies or NETs, from DNA selectively packaged in extracellular vesicles?

Which method is suitable to enrich homogenous EV-containing DNA for diagnostic and functional studies?

Comparable to real-time monitoring of species in natural ecosystems using EV-DNA, how sensitive and reliable would EV-DNA be in monitoring the spatiotemporal status of intratumor heterogeneity in cancer evolution?

What are the mechanisms and intracellular conditions responsible for DNA packaging in extracellular vesicles?

Are there specific EV biogenesis pathways, as yet unexplored, and sorting mechanisms responsible for packaging EV-DNA into EVs? Or are there specific mechanisms for loading different EV-DNA types (e.g., mtDNA or gDNA) in specific EV populations?

How is EV-DNA taken up by the recipient cells in the tumor microenvironment, and what are the downstream functional responses triggered for sensing this extracellular DNA?

Does the gDNA/mtDNA packaging in vesicles affect its organization and its fate in recipient cells?

How does tumor-derived EV-DNA hijack cytoplasmic defense in recipient cells to conquer the nucleus causing genetic/epigenetic transformation?

## Data Availability

Not applicable.

## Abbreviations

dsDNA: Double-stranded DNA
ssDNA: Single-stranded DNA
EVs: Extracellular vesicles
LEVs: Large extracellular vesicles
SEVs: Small extracellular vesicles
EPs: Extracellular particles
MVs: Membrane vesicles
OMVs: Outer membrane vesicles
IOMVs: Inner outer membrane vesicles
EV-DNA: Extracellular vesicles DNA
cfDNA: Cell-free DNA
exDNA: Extracellular DNA
eDNA: Environmental DNA
mtDNA: Mitochondrial DNA
HGT: Horizontal gene transfer
ESCRT: Endosomal sorting complex required for transport
MN: Micronuclei
cGAS: Cyclic GMP-AMP synthase
STING: Stimulator of interferon genes
DSB: Double-strand break
